# Pathophysiology of Disseminated Intravascular Coagulation in Sepsis: A Clinically Focused Overview

**DOI:** 10.3390/cells12172120

**Published:** 2023-08-22

**Authors:** Ahsanullah Unar, Lorenzo Bertolino, Fabian Patauner, Raffaella Gallo, Emanuele Durante-Mangoni

**Affiliations:** 1 Department of Precision Medicine, University of Campania ‘L. Vanvitelli’, 80138 Naples, Italy; ahsanullah.ahsanullah@unicampania.it (A.U.); lorenzo.bertolino@unicampania.it (L.B.); fabianpatauner@gmail.com (F.P.); raffaella.galloo@gmail.com (R.G.); 2 Unit of Infectious and Transplant Medicine, AORN Ospedali dei Colli-Monaldi Hospital, 80131 Naples, Italy

**Keywords:** sepsis, disseminated intravascular coagulation, platelets, mechanism, thrombosis, therapy, pathogenesis, immune cell, treatment

## Abstract

Sepsis is a major global health problem that results from a dysregulated and uncontrolled host response to infection, causing organ failure. Despite effective anti-infective therapy and supportive treatments, the mortality rate of sepsis remains high. Approximately 30–80% of patients with sepsis may develop disseminated intravascular coagulation (DIC), which can double the mortality rate. There is currently no definitive treatment approach for sepsis, with etiologic treatment being the cornerstone of therapy for sepsis-associated DIC. Early detection, diagnosis, and treatment are critical factors that impact the prognosis of sepsis-related DIC. Over the past several decades, researchers have made continuous efforts to better understand the mechanisms of DIC in sepsis, as well as improve its quantitative diagnosis and treatment. This article aims to provide a comprehensive overview of the current understanding of sepsis-related DIC, focusing on common causes and diagnoses, with the goal of guiding healthcare providers in the care of patients with sepsis.

## 1. Introduction

Sepsis and disseminated intravascular coagulation (DIC) are interrelated conditions that pose a major threat to global health [1]. In 2001, the International Society for Thrombosis and Hemostasis revised its definition of DIC. DIC is traditionally classified as consumptive coagulopathy, given that its diagnostic criteria are centered around the occurrence of decompensated coagulopathy. However, the definition of DIC also includes the systemic activation of coagulation and endothelial dysfunction, which are integral to its pathophysiology. To reconcile these aspects, the development of disease-specific criteria is underway to enhance both diagnosis and management. In the context of sepsis-associated DIC, innovative strategies, such as a two-step diagnostic process using sepsis-induced coagulopathy (SIC) and the incorporation of new biomarkers, are being considered. As research advances, the need to continually refine our understanding of DIC’s specific implications through both laboratory and clinical research remains paramount (Table 1) [2,3].

The definition of sepsis has evolved over time, with the latest international consensus defining it as organ failure that results from a dysregulated host response to infection [4]. Although rational antibiotic therapy can control the underlying infection, once triggered, the uncontrolled host response continues to persist, resulting in the high morbidity and mortality observed in sepsis and septic shock (Table 1) [11].

Sepsis is a life-threatening condition that results from a widespread immune-inflammatory response to infection, while DIC is a secondary complication that occurs in up to 80% of patients with sepsis. It is characterized by the systematic activation of the coagulation cascade, leading to the formation of thrombi within the vasculature, with a particular predilection for smaller blood vessels, such as capillaries and organ damage [6]. The interrelation of these conditions highlights the complex nature of bacterial infections and their impact on the body’s immune and clotting systems. Additionally, patients with underlying thrombophilia conditions, characterized by an increased tendency for blood clot formation, are at high risk of developing DIC. This is due to their already primed coagulation system, a status that can be exacerbated by sepsis [12,13]. This assertion is supported by a body of experimental evidence. For instance, Langerak et al. [14] suggest that individuals with variations in the regulation of their procoagulant, anticoagulant, and fibrinolytic systems, such as those with thrombophilia, may be exposed to additional risk factors for DIC [14]. Furthermore, Prazanowski et al. [15] found that activated protein C resistance (APCR), a genetically determined cause of thrombophilia, can be a risk factor for DIC. Yildirim et al. [16] also suggest that patients with thrombophilia have higher risk scores for conditions such as sepsis-induced coagulopathy (SIC) and DIC. Table 1 provides a comprehensive delineation of the pathology, mechanisms, clinical manifestations, diagnostic markers, and therapeutic interventions associated with SIC, DIC, and related conditions. The correlation of these conditions with the contentious Sepsis-3 definition, in conjunction with the latest research findings, is further elucidated in Table 2. Moreover, Hofstra et al. [17] and Tikkanen et al. [18] both support the notion that thrombophilia can be a risk factor for DIC, with the latter suggesting that the combination of hyperhomocysteinemia and thrombophilia increases the risk of DIC. Therefore, the interrelation of these conditions and the role of thrombophilia as a risk factor for DIC is well supported by the current body of research [14,15,16,17]. The mechanisms underlying the relationship between sepsis and DIC are not fully understood. However, researchers have proposed several theories to explain this relationship. One theory suggests that DIC is a direct result of an underlying infection, particularly in cases of IE [19]. The formation of vegetation on heart valves can result in valve dysfunction and the spread of bacteria throughout the bloodstream, leading to sepsis and DIC [20,21]. Another theory proposes that DIC occurs because of the release of cytokines and other signaling molecules in response to the initial infection. These signaling molecules activate the clotting cascade and lead to the formation of clots [20,22]. Studies have shown that prompt diagnosis and treatment of infection are critical to reduce the risk of sepsis and DIC and improve patient outcomes. The use of antibiotics and other anti-infective agents is the cornerstone of therapy for sepsis-related DIC, with early detection and treatment being critical factors that impact the prognosis of patients with this condition [23,24].

In recent years, researchers have made significant advances in the understanding of sepsis-related DIC, including its causes, diagnosis, and management. For example, the development of new diagnostic tools, such as the use of biomarkers for the early detection of DIC in sepsis, has improved the ability to diagnose and treat sepsis-related DIC [39,40].

Overall, sepsis, DIC, and thrombophilia appear to be interrelated conditions that pose a major threat to health. The interrelation of these conditions highlights the complex nature of bacterial infections and their impact on the body’s immune and clotting systems [41,42]. To better understand the mechanisms underlying the relationship between sepsis and DIC, we reviewed the literature with a focus on evidence-based effective therapeutic strategies.

## 2. Understanding the Role of Bacterial Virulence in the Pathogenesis of Sepsis and Associated Conditions

Researchers have examined the microbiological features of several sepsis-causing bacteria that pose challenges to host defense [43]. A group of bacteria, including *Staphylococcus aureus*, *coagulase-negative staphylococci (CoNS)*, *Streptococcus pneumoniae*, *Haemophilus influenzae b*, *Neisseria meningitidis*, *Klebsiella pneumoniae*, *Enterococcus faecalis*, *Acinetobacter baumanii*, *Escherichia coli*, *Salmonella enterica*, *Shigella dysenteriae*, *Citrobacter freundii*, *Serratia marcescens*, *Proteus mirabilis*, *Pseudomonas aeruginosa*, and *Bacteroides fragilis*, are implicated in severe systemic infections, such as sepsis, DIC, SIC, and septic shock [44,45]. The controversy surrounding these bacteria lies not in their disease-causing potential but in the mechanisms, they employ and the variability in their pathogenicity. Each bacterium possesses a unique set of virulence factors, and the host’s immune response to these factors can significantly influence the disease outcome [44,45]. For example, *S. aureus* produces toxins and biofilms, damages host tissues and immune cells, and provides protection from immune responses and antibiotics [44]. *CoNS*, often overlooked, have emerged as significant pathogens in immunocompromised individuals and those with implanted medical devices [45]. *S. pneumoniae* and *H. influenzae b*, known for their polysaccharide capsules, evade phagocytosis [46]. *N. meningitidis* can invade the bloodstream, causing sepsis and DIC, with its endotoxin triggering a massive inflammatory response, leading to septic shock [45]. Enterobacteriaceae, including *K. pneumoniae*, *E. coli*, *S. enterica*, *S. dysenteriae*, *C. freundii*, *S. marcescens*, and *P. mirabilis*, possess various virulence factors, causing a range of infections and, in severe cases, sepsis and septic shock [44,45].

*E. faecalis*, *A. baumanii*, *P. aeruginosa*, and *B. fragilis*, part of the normal human microbiota, can become opportunistic pathogens, causing infections that can progress to sepsis and septic shock if untreated [44,45]. The controversy lies in the complex interplay between these bacteria and the host’s immune system [44]. The host’s immune response plays a significant role in determining disease outcomes, with an overactive response leading to a ‘cytokine storm’, causing conditions such as sepsis, DIC, and septic shock [43,45]. Table 3 provides a list of bacteria that cause sepsis and their microbiological features that pose problems to the host defense [43].

Overall, the pathogenesis of sepsis, DIC, and septic shock involves both bacterial virulence factors and the host’s immune response. The variability in these factors makes understanding this process challenging, and it remains a subject of ongoing research and debate [43,44,45].

## 3. Inflammatory Mediators Associated with Pyroptosis and Their Role in Subsequent Coagulation Disorders

**Figure 1:**
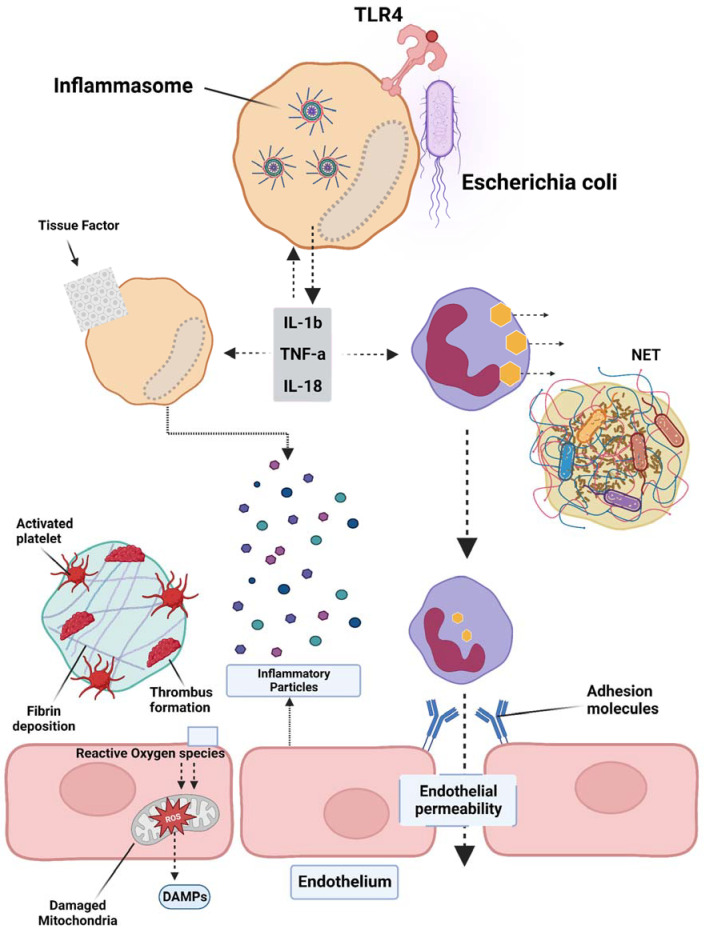
Overview of the pathogenesis of sepsis, which involves several pathophysiological processes, such as endothelial injury, breakdown of the endothelial barrier, immune thrombosis, and disseminated intravascular coagulation (DIC). Various factors contribute to the development of sepsis, including damage-associated molecular patterns (DAMPs), interleukins (ILs), Toll-like receptor 4 (TLR4), and tumor necrosis factor-alpha (TNF-α). The interplay of these factors in the pathogenesis of sepsis leads to the activation of coagulation pathways and inflammatory responses, and organ and organ dysfunction.

Sepsis is a condition caused by infection, with Gram-negative bacterial infections being the most prevalent, accounting for approximately 60% of cases, while Gram-positive bacterial infections account for approximately 40%. When pathogenic microorganisms invade the body, they can be swiftly recognized by the pattern recognition receptors (PRRs) of immune cells, leading to a series of inflammatory responses [47]. PRRs are located both on the cell membrane and intracellularly, with Toll-Like Receptor 4 (TLR4) being the most well-studied membrane PRR capable of recognizing lipopolysaccharides (LPS) in Gram-negative bacteria. The inflammasome is a receptor complex that directly detects the presence of pathogenic microorganisms within the cytoplasm. Cytokine storms resulting from excessive activation of the TLR4 receptor by extracellular LPS are the primary cause of DIC. The TLR4 receptors on immune cell membranes, with the aid of coreceptors Myeloid Differentiation Factor 2 (MD2) and Cluster of Differentiation 14 (CD14), recognize highly conserved lipids within extracellular LPS. Signals are conveyed to the cell through Myeloid Differentiation primary response 88 (MyD88), and TIR-domain-containing adapter-inducing interferon-β (TRIF) activates the Nuclear Factor kappa-light-chain-enhancer of activated B cells (NF-κB), interferon regulator 3, and other factors, thereby promoting the transcription and secretion of cytokines, chemokines, and other mediators. The activation of the inflammasome thus results in the maturation, release, and pyroptosis of interleukin-1β (IL-1β) and interleukin-18 (IL-18). Pyroptosis is a proinflammatory form of programmed death discovered in recent years that depends on caspases (*casp1* and *casp 4/5* or *casp11* in mice), leading to the cleavage of the N-terminus of the Gasdermin D (GSDMD) protein. Activated GSDMD causes holes in the cell membrane, leading to osmotic swelling, cell death, and the release of large amounts of inflammatory contents, further exacerbating the inflammatory response [48,49]. Studies have shown that mice with *tlr4* knockouts and defects in *Casp1* (*Casp1^−/−^*) or *Casp11* (due to *Casp1* chromosome defects) in neighboring encoding genes have demonstrated tolerance to high doses of LPS [50,51]. Previous research found that *Casp1^−/−^* mice from the 129-mouse background were *Casp1* and *Casp11* double-knockouts, as confirmed by immunoblotting protein analysis. The immunoblotting results indicated that the *Casp1* and *Casp11* double-knockout mice derived from the 129-background were protein deficient, except for the mice in [52,53]. A study by Kayagaki’s team found that LPS, with the aid of cholera toxin B (CTB) or certain Gram-negative bacteria, could cause wild-type *C57BL/6* mouse BMMs to release IL-1β and trigger pyroptosis, which was not observed in the 129-background mice. C57BL/6 background-derived *Casp11^−/−^* mice were found to resist high doses of LPS, while *Casp1^−/−^* mice with only the *Casp1* deficiency could not resist LPS, indicating that the LPS-induced activation of the *Casp11* signaling pathway plays a crucial role in sepsis pathology. Further research revealed that only intracellular LPS can activate *Casp11* independently of TLR4 [53,54,55]. Although much research has been performed in recent years on the role of inflammasome activation in sepsis pathology and poor prognosis, the specific mechanism is still not understood. A recent study by Wu et al. elucidated the pivotal role of macrophage pyroptosis in sepsis. During pyroptosis, macrophages undergo cell membrane rupture, leading to the release of tissue factor-containing microparticles (TF MPs). These TF MPs initiate and amplify the extrinsic coagulation cascade, leading to organ failure and DIC, ultimately resulting in the death of the septic host [56]. The release of TF MPs from macrophages is contingent on cell lysis, which is dependent on GSDMD. Macrophages deficient in GSDMD do not exhibit the release of TF MPs upon stimulation. Conversely, the release of TF MPs from wild-type macrophages can be inhibited using a cell membrane stabilizer [36]. Wu et al. further substantiated that monocytes/macrophages are the primary sources of TF MPs in sepsis by employing conditional knockout mice and depleting the monocytes/macrophages pharmacologically [56]. Pyroptosis of cells, accompanied by the release of substantial amounts of inflammatory mediators, also plays a significant role in subsequent coagulation disorders (Figure 1).

## 4. Platelet Activation and Its Effects on the Coagulation Cascade in Sepsis

TF is a key player in the development of sepsis-related DIC, a pathological condition characterized by abnormal coagulation. Inhibiting TF activity through drugs or gene deletion has been shown to reduce coagulation disorders and decrease mortality in mice with sepsis [57]. TF is expressed in various cells surrounding blood vessels, such as pericytes, fibroblasts, and vascular smooth muscle cells, as well as in blood vessels themselves [58]. Additionally, activated cells, such as endothelial cells, neutrophils, and eosinophils, were previously believed to express TF; however, further research showed that these cells acquire TF from monocyte-derived MPs through surface receptors [58].

The expression of TF on platelets is still a topic of debate, with some studies suggesting that TF can be transferred to the platelet surface after activation and others showing conflicting results. Platelets, in both resting and activated states, have been shown to express varying levels of TF at the mRNA and protein levels; however, this observation has not been consistently supported by all research teams [58]. Activated platelets can also be derived through their surface P-selectin or through binding with monocyte MPs or CD15 PSGL-1 (P-Selectin Glycoprotein Ligand-1), which may contribute to the expression of TF on the platelet surface [58]. In a study conducted by Pawlinski’s team, a sepsis model was engineered using conditional knockout mice [58]. The investigators documented a significant decrease in thrombin-antithrombin complex (TAT) levels 8 h after LPS exposure in mice where tissue factor (TF) was genetically ablated in either hematopoietic or nonhematopoietic cells [58]. This pattern was also evident in mice with TF ablation in myeloid cells or in a combination of endothelial and hematopoietic cells. However, the targeted genetic ablation of TF in endothelial cells or vascular smooth muscle cells did not significantly alter the plasma TAT levels in septic mice. These findings underscore the critical role of TF, originating from both myeloid cells and an as-yet-unidentified nonhematopoietic cell source, in instigating the coagulation cascade during sepsis [58]. Although the in vitro evidence shows that endothelial cells can express a high level of TF, the in vivo experiments have not consistently found a positive expression of TF on the endothelium. The conditional knockout of TF in endothelial cells also did not have a significant impact on coagulation activation in septic mice. This makes endothelial cells unlikely to be a main source of TF expression or release [59].

**Figure 2:**
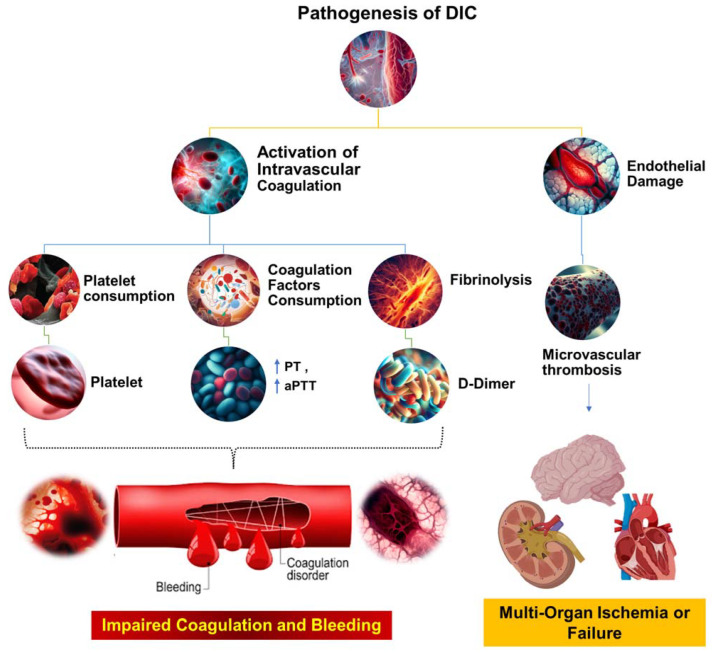
The pathophysiological mechanisms underlying DIC. This figure illustrates the complex cascade of events that occur during the pathogenesis of DIC. The process begins with systemic activation of the coagulation system, which results in extensive fibrin production and deposition. These fibrin deposits then create microvascular thrombi, which can impede blood flow and cause multiorgan dysfunction. Concurrently, significant coagulation activity consumes critical hemostatic components, such as clotting factors and platelets. This depletion can upset the delicate balance of hemostasis, potentially leading to severe, life-threatening bleeding. Prothrombin time (PT); aPTT (activated partial thromboplastin time).

Wu et al. recently found that by using chlorophosphate liposomes to deplete nearly 90% of the monocytes and macrophages, plasma TAT levels in septic mice were significantly reduced, leading to a >50% increase in survival. This suggests that monocyte/macrophage-derived TF is a major contributor to the activation of sepsis coagulation [56]. Previous studies have shown that systemic proinflammatory cytokines resulting from infection cause the overexpression of TF in monocytes/macrophages [60]. Numerous animal models of sepsis and clinical studies in sepsis patients have shown a significant increase in the number of circulating TF-positive MPs of monocyte/macrophage origin, which is strongly linked to coagulation activation, organ failure, and death. However, the mechanism behind the formation of these circulating soluble TF MPs has only recently been uncovered. Wu et al. found that proteins from the type III secretory system of bacteria and LPS activate small classical and nonclassical inflammatory components, respectively. Gasdermin D causes macrophage pyroptosis in vivo but does not release TF MPs from cell membrane-bound wells. This process relies on Gasdermin D-mediated osmotic membrane cleavage, which can be significantly reduced with the formation of TF MPs. In sepsis, microvascular damage and the onset of DIC are interrelated. Endothelial cells are critical targets of attack by danger-associated molecular patterns (DAMPs) and inflammatory agents [61]. Normally, endothelial cells exert anticoagulant and anti-inflammatory effects; however, upon inflammatory stimulation, exposure to TF in the subendothelial layer triggers exogenous coagulation activation. At the same time, endothelial cells initiate a series of procoagulant and proinflammatory processes by upregulating adhesion molecule expression and attracting and activating immune cells such as monocytes and neutrophils. Secretion of the von Willebrand factor (vWF) also promotes platelet aggregation and platelet-dependent coagulation [61]. In sepsis, platelets can be activated by DAMPs, inflammatory mediators, thrombin, and vWF, resulting in increased expression of activated platelet P-selectin, which boosts monocyte TF expression by binding to the PSGL-1 receptors on the surface of monocytes [46,62]. Activated platelets provide an ample phospholipid surface that significantly amplifies coagulation cascade reactions while reducing blood protease inhibitors, thus inhibiting enzymes in the coagulation reactions [59]. Once activated, platelet-dense granules release soluble polyphosphate to their surface, triggering factor XII (FXII) formation and promoting thrombin production via the FXII pathway (Figure 2) [63]. 

In recent years, extensive research has been conducted to understand the role of platelets in the pathogenesis of sepsis, revealing them as essential bridges connecting the hemostatic/coagulation system with the immune system [64,65]. Platelets exhibit complex interactions with bacteria during infection, constituting an important part of the immune response and thrombus formation [66]. Studies have shown that platelets are among the first cells to be activated during sepsis [47]. Activated platelets express upregulated markers, such as P-selectin, CD63, and CD61, and form aggregates with neutrophils and monocytes [67]. Researchers, such as Soriano et al., have measured platelet-derived microparticles and platelet-leukocyte aggregates in sepsis patients, reporting a strong correlation between these markers and disease severity [68]. Platelet count reduction is common in sepsis patients, with the degree of reduction correlating with disease severity, and persistent thrombocytopenia is an independent predictor of poor prognosis [69]. Increased platelet isolation and consumption in organs such as the lungs and liver contribute significantly to thrombocytopenia in sepsis [69,70].

**Figure 3:**
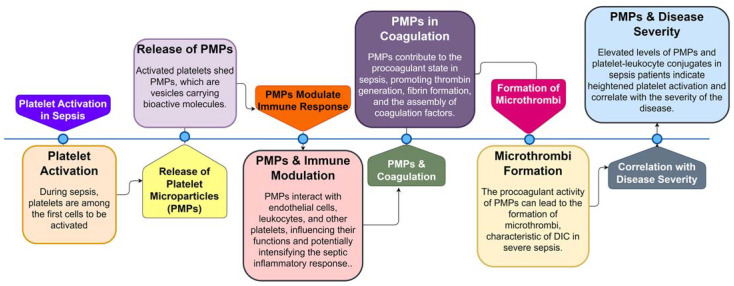
Sequential Role of Platelets and Platelet Microparticles (PMPs) in Sepsis Pathogenesis. This flowchart illustrates the step-by-step involvement of platelets and PMPs during sepsis, from initial platelet activation to the formation of microthrombi and the correlation of PMP levels with disease severity.

Platelet microparticles (PMPs) are small vesicles released from activated platelets during sepsis. These PMPs carry a variety of bioactive molecules, including procoagulant factors, cytokines, and microRNAs, making them potent mediators of coagulation and inflammation. PMPs are involved in the formation of microthrombi, contributing to the procoagulant state in sepsis (Figure 3) [65,71,72,73,74]. Additionally, PMPs can interact with endothelial cells, leukocytes, and other platelets, further amplifying the inflammatory response and potentially exacerbating organ damage in sepsis.

## 5. Tissue Factor Pathway Inhibitor (TFPI) and Fibrin Deposition during Sepsis

The activation of the coagulation pathway during sepsis is accompanied by the inhibition of the three main anticoagulant systems: protein C (PC), AT, and TFPI. In sepsis, the levels and activity of AT, TFPI, and PC are significantly reduced in both animals and patients. Under normal conditions, the binding of thrombin to thrombomodulin (TM) on endothelial cell membranes increases the activation rate of PC by 100-fold while also inhibiting various thrombin functions, such as the binding of fibrinogen to fibrin and the binding of thrombin to platelet and immune cell receptors [63]. Activated PC (APC) exerts anticoagulation by hydrolyzing the cofactors Va and VIIIa and enhances PC activation by binding to the endothelial protein C receptor (EPCR) and the thrombin complex [75]. Studies in animals have shown that inhibiting the PC system or having heterozygous mutations in the *PC* gene significantly increases the mortality of sepsis-induced DIC, whereas APC supplementation can improve organ function and prognosis [46,76]. Despite PC supplementation, low levels of APC in the plasma of some sepsis patients indicate impaired PC activation in the body. Plasma APC levels are variable in sepsis patients, and lower levels are associated with a poor prognosis [77]. The in vitro experiments have shown that inflammatory mediators can decrease endothelial cell TM and endothelial protein C receptor (EPCR) expression; however, there is conflicting evidence from animal studies [46]. Plasma concentrations of soluble Thrombomodulin (sTM) and EPCR in sepsis patients and animals increase significantly, and high levels of sTM are strongly correlated with the severity and prognosis of the disease [46]. TFPI is a primary inhibitor of the TF/Factor VIIa (FVIIa) complex and Factor Xa (FXa) and administering TFPI antibodies increases fibrin deposition in the lungs of septic animals [78]. The anticoagulant effect of TFPI requires further investigation.

## 6. Thrombin-Activated Fibrinolysis Inhibitor (TAFI) and Its Role in Sepsis

The inhibition of fibrinolysis is a crucial aspect of septic disseminated intravascular coagulation (DIC). In sepsis, the production of Plasminogen Activator Inhibitor-1 (PAI-1) and Tissue Plasminogen Activator (t-PA) by the endothelium increases dramatically, however, PAI-1 significantly exceeds t-PA, resulting in the inhibition of fibrinolysis [79]. Notably, the in vitro experiments have demonstrated that thrombin liberates PAI-1 from human liver endothelium, a process followed by de novo synthesis [80]. Patients with sepsis exhibit persistently elevated levels of plasma PAI-1, and the greater the degree of fibrinolytic inhibition, the more severe the illness [62]. Recent studies have uncovered other mechanisms of thrombin-dependent fibrinolytic inhibition, such as thrombin strengthening the clot to increase resistance to fibrinolysis and activating thrombin-activated fibrinolysis inhibitor (TAFI), thus decreasing plasmin production [81]. TAFI, a single-chain glycoprotein produced by the liver, is secreted into plasma and regulates fibrinolysis when activated by thrombin. Thrombin binding to TM enhances TAFI activation [81]. In sepsis, TAFI levels in plasma tend to be reduced in patients due to activation or depletion. Inhibiting thrombin-TM-dependent TAFI activation enhances fibrin degradation and reduces tissue fibrin deposition. Elevated levels of TAFI activation markers are found in the plasma of DIC patients and deceased patients compared to those without DIC and those who survive, and these elevated levels are strongly correlated with the severity of the disease [59]. Platelets activated by αIIbβ3 (integrin alpha-IIb/beta-3, also known as glycoprotein IIb/IIIa (GPIIb/IIIa)) can inhibit fibrinolysis by directly binding to fibrin, altering its structure, and activating TAFI [59].

## 7. Epigenetic Alterations and Immunosuppressive Immune Cell Phenotypes

In recent years, the intricacy of the host’s response in sepsis has been better understood. Sepsis is a result of multiple responses that include persistent excessive inflammation, immunosuppression, and the imbalance of homeostasis. Immunosuppression in sepsis is marked by the depletion of lymphocytes and the rearrangement of antigen-presenting cells. In sepsis, large quantities of Cluster of Differentiation 4 (CD4^+)^ and CD8^+^ T lymphocytes, B lymphocytes, and dendritic cells (DCs) are lost due to apoptosis. Preventing lymphocyte apoptosis through drugs or genetic methods has shown significant improvement in the prognosis of septic animals [11,82,83]. In sepsis, CD4^+^ T-helper cells, specifically Th1, Th2, and Th17 cells, are suppressed. Experiments involving the extraction of lymphocytes from sepsis patients have revealed that the ability of the T lymphocytes to secrete Interferon gamma (IFNγ) and Tumor Necrosis Factor (TNF) in the spleens of deceased sepsis patients was significantly reduced, while their expression of programmed cell death protein 1 (PD-1) was significantly increased, along with PD1 ligand 1 (PDL1) on macrophages and endothelial cells. Inhibiting the PD1-PDL1 axis reduced mortality in mice with sepsis, proving that the interaction of PD1-PDL1 is a mechanism of cell depletion. Regulatory T cells (Tregs), which can inhibit monocyte and neutrophil function, are significantly increased in sepsis, and blocking their function can improve immune function and bactericidal ability [82,83,84,85]. In sepsis, the expression of Human Leukocyte Antigen - DR isotype (HLA-DR (MHC class II cell surface receptor)) on the surface of monocytes and DCs is reduced, and the ability of monocytes/macrophages to secrete proinflammatory cytokines is reduced when LPS stimulation is administered in vitro, a phenomenon known as immunoparalysis or LPS tolerance. Patients with sepsis have increased DC apoptosis, and inhibiting DC apoptosis can improve the prognosis of septic mice. Recent discoveries have shown that epigenetic alterations can result in immunosuppressive immune cell phenotypes. For example, LPS-induced tolerance is associated with decreased levels of monocyte histone H3 lysine 4 trimethylation, and IL-1β in macrophages has increased levels of histone H3K9 (ninth lysine (K) residue of the histone H3 protein) dimethylation in the promoter region of the (Tumor Necrosis Factor alpha) *TNF-α* gene [86].

## 8. The Role of Extracellular Nuclear Products in Sepsis, Coagulation Disorders, and Thrombosis

In recent years, researchers have discovered that extracellular nuclear products play a critical role in sepsis, coagulation disorders, and thrombosis. These products include neutrophil extracellular traps (NETs), extracellular histones, and high mobility group protein B (HMGB1). NETs are structures released by neutrophils in response to stimulation (such as microorganisms, inflammatory mediators, and reactive oxygen species) and contain a range of substances, including elastase, myeloperoxidase, histones, and Deoxyribonucleic Acid (DNA), which have broad bactericidal effects [87]. Other immune cells, such as mast cells, eosinophils, and mononuclear phagocytes, can also release NETs after activation [88]. Histones, which are basic small molecule proteins bound to DNA in chromosomes, can be released from cells (primarily neutrophils) when they are damaged or dead and exist in the circulation as histone-DNA complexes (nucleosomes) or as part of NETs. They are the most abundant proteins in NETs [89]. There is a feedback loop between extracellular histones and NETs, as histones can stimulate the neutrophil release of NETs that contain histones or modified histones [89].

HMGB1 is a highly conserved protein that can be actively secreted by stimulated immune cells or passively released by necrotic cells and acts as a late, lethal proinflammatory factor [90]. This is seen in both animal models and patients’ extracellular histones, NETs, and HMGB1, which have been shown to play a significant role in thrombosis, organ failure, and poor prognosis in recent years [89,90,91,92,93]. NETs, particularly their DNA and nucleosomes, can activate both the exogenous and endogenous coagulation pathways through interaction with TF MPs and activation of factor XI and FXII under pathological conditions [94,95]. Extracellular histones can directly activate the NF-κB and Activator Protein 1 (AP-1) pathways through TLR4 and TLR2 receptors on the surface of endothelial cells and macrophages, leading to an upregulation of TF expression [96]. Additionally, direct activation of the endogenous coagulation system can also trigger autologous activation of factor I and thrombin-mediated activation of XI by promoting platelet polyphosphate release [74]. Extracellular histones and NETs can promote the release of inflammatory mediators such as IL-6, IL-1β, and TNF-α by monocytes and macrophages, further disrupting the coagulation-anticoagulation-inflammatory balance and leading to the amplification of the procoagulant phenotype [11]. Platelet activation, either directly or indirectly, by histones and NETs; can promote thrombin production, and activated platelets can in turn promote histone release and NETs [97]. Histones and NETs can also inhibit the anticoagulant system through multiple pathways. For example, histones can downregulate TM and inhibit protein C activity, and elastase in NETs can directly degrade AT and TFPI, leading to decreased anticoagulant synthesis by the liver and increased leakage into the tissue space [89]. Additionally, histones and NETs can downregulate plasminogen activation by t-PA, inhibiting fibrinolysis [98]. The level of circulating histones in patients with sepsis is closely correlated with the endothelial damage marker sTM, and in vitro experiments have shown that extracellular histones have direct toxicity to vascular endothelial cells, causing damage and exposing subendothelial collagen and TF, promoting the occurrence of coagulation reactions [99].

Endothelial cells have a limited ability to phagocytize NETs, which can lead to disruption of their tight junctions and dysfunction. In turn, activated endothelial cells contribute to NET formation [100,101]. The cellular damage caused by histones and NETs can result in exposure to highly procoagulant phospholipids, amplifying prothrombin reactions by up to 25,000 times [102]. In animal models of sepsis, the in vivo injection of histones has been shown to cause endothelial injury, alveolar hemorrhage, microvascular thrombosis, and even death. However, administration of histone antibodies can alleviate these symptoms [93]. Similarly, inhibiting NET formation can prevent the formation of thrombi [103].

HMGB1 has been found to stimulate TF expression in monocytes and macrophages while reducing the activity of PC by inhibiting the thrombin-TM complex [104,105]. Platelet-derived HMGB1 has also been shown to promote NETs through advanced glycosylated end-product receptors [106]. Using conditional knockout mice, Deng’s team discovered that mainly liver-derived HMGB1 delivers extracellular LPS to the macrophages and endothelial cells, leading to *Casp11* activation [107]. Wu et al. demonstrated that pyroptotic macrophages, through the secretion of TF MPs, can cause DIC and eventually lead to the death of mice with sepsis [56]. Inhibition of HMGB1 has been shown to significantly improve the prognosis in septic animals [104].

In summary, histones, NETs, HMGB1, and other factors can contribute to the development of DIC through various pathways. The pathological mechanism of sepsis DIC is complex and involves intertwined factors such as inflammation, coagulation, and immunity.

## 9. Unraveling the Interplay of Sepsis, SARS-CoV-2, and Flaviviruses: A Comparative Analysis of Molecular Pathogenesis and Controversial Therapeutic Implications

The intricate interplay of immune responses and viral characteristics in sepsis, SARS-CoV-2, and flaviviruses forms a complex molecular pathogenesis. This study aims to discern the potential of these viruses as contentious sepsis triggers and to ascertain the similarities or disparities in their molecular pathogenesis Such an understanding is pivotal for shaping future research and the therapeutic strategies for these prevalent diseases (Table 1 and Table 2). Sepsis, an immune response elicited by infection, triggers a “cytokine storm,” leading to a hyperinflammatory state and the release of numerous cytokines across various tissues [34]. SARS-CoV-2 infiltrates human cells via ACE2 receptors, inducing a potent immune response and lung inflammation after rapid replication [34,108,109]. The role of ACE2 extends beyond viral entry, influencing blood pressure and inflammation regulation [34,110,111]. Flaviviruses, upon replication within endothelial cells, monocytes, and dendritic cells, release pro-inflammatory cytokines and chemokines, potentially leading to sepsis-like syndromes. The clinical manifestations of flavivirus-induced immune dysregulation, such as dengue’s hemorrhagic fever or Zika’s neurological complications, add complexity to understanding these viruses [9]. While sepsis, SARS-CoV-2, and flaviviruses share similarities in immune response triggering, the specific mechanisms and outcomes vary significantly. For instance, SARS-CoV-2 and influenza viruses can cause severe lung damage, leading to conditions such as ARDS, a common sepsis outcome [34,36,110,111].

The potential of these viruses as sepsis triggers remains controversial. Some studies suggest a strong link between viral infections and sepsis, while others dispute this. For instance, the reactivation of a single virus does not significantly increase sepsis mortality, but the reactivation of multiple viruses may exacerbate sepsis [5,6,7,8,33,112].

In conclusion, while sepsis, SARS-CoV-2, and flavivirus share molecular pathogenesis similarities, significant differences exist in the molecular pathology of flavivirus (refer to Table 2). The potential of these viruses as sepsis triggers remains a contentious topic requiring further research. Future studies should address key unresolved questions, including organ contributions to sepsis progression, the influence of SARS-CoV-2 and ACE2 receptor interactions on COVID-19 pathogenesis, the mechanisms of flavivirus-induced immune dysregulation, the differences in immune responses to these viruses, the relationship between viral reactivation and sepsis progression, and the development of effective treatments. Interdisciplinary research is crucial to advancing our understanding and treatment of these conditions.

## 10. Research Progress in the Treatment of Sepsis-related DIC

The treatment of sepsis-related DIC has primarily focused on antimicrobial therapy, including surgical drainage of any infected site; and symptomatic supportive care, such as alternative therapy and fluid resuscitation [113]. With a deeper understanding of the pathophysiological mechanisms of sepsis-related DIC, anticoagulation therapy and new treatment methods are being explored. The 2013 ISTH guidelines recommend the use of unfractionated heparin (UFH) over low molecular weight heparin (LMWH) for the treatment and prevention of thrombosis; however, there is limited high-quality evidence to support this recommendation [114]. Some small, randomized controlled trials have shown that low-dose heparin may improve hypercoagulability and prognosis in early-stage sepsis patients but not necessarily in those with sepsis DIC [115]. A subgroup analysis of a phase III clinical trial found that recombinant solubility TM (rsTM) was more effective than UFH in alleviating DIC and reducing mortality in patients with infectious DIC. Another small randomized, double-blind; clinical trial found no significant difference in the response rates between UFH and APC for DIC; however, reduced bleeding risk and mortality. The use of heparin in sepsis DIC remains controversial, and further high-quality randomized controlled trials are needed to reach a conclusion [27,115,116].

APC, an anticoagulant and anti-inflammatory agent, also has the ability to degrade extracellular histones [117]. The efficacy of recombinant APC was tested in the PROWESS clinical trial; here, subgroup analysis revealed that rAPC improved the prognosis of sepsis patients with DIC [118]. Based on these findings, international guidelines recommended the use of recombinant APC for sepsis DIC patients in 2011 [119]. However, a subsequent 2012 RCT showed that recombinant APC increased the risk of bleeding and did not reduce mortality in sepsis or septic shock patients, leading to its withdrawal from the market. Despite this, a small, randomized, controlled trial demonstrated that plasma-derived APCs significantly reduced mortality in DIC patients, however, further validation is necessary [120]. In a large phase III clinical trial, high-dose AT therapy failed to reduce mortality in sepsis patients and increased the risk of bleeding [121]. Subgroup analysis showed that high-dose AT reduced sepsis-related mortality in DIC patients without significant bleeding events [121]. On the other hand, it significantly increased the risk of bleeding in patients without sepsis and DIC. Several meta-analyses and large observational studies have supported the use of AT supplementation in sepsis DIC patients [122,123]. In Japan, AT supplementation is recommended in sepsis DIC patients with decreased AT activity. However, this treatment is not widely adopted outside of Japan [124].

The recommendation of use for rsTM in sepsis is currently in place in Japan. However, the latest SCARLET trial, a phase III clinical trial, has reported that rsTM does not significantly lower the mortality rate of patients with sepsis coagulopathy, although not all coagulation disorders included reached the level of DIC [125]. A meta-analysis incorporating the latest SCARLET results showed that rsTM treatment reduced mortality in sepsis and coagulopathy by approximately 13%, although it was not statistically significant. Additionally, the treatment did not increase the risk of bleeding in patients [125]. The effectiveness of rsTM in patients with sepsis DIC remains unclear, and further high-quality RCTs are needed.

Inflammation plays a significant role in sepsis DIC, with monoclonal antibodies showing promise in animal models. However, human clinical trials have been disappointing due to their limitations. Polymyxin B hemoperfusion, a blood purification procedure, has shown an improvement in hemodynamic and organ dysfunction in septic shock patients, however, further trials are needed to determine its effectiveness and the role of LPS levels in patients [126,127].

Immunosuppression is a critical aspect of sepsis, and reversing immune function with drugs such as IFNγ, IL-7, and IL-15 and antibodies against suppressive immune checkpoints may reduce secondary infections and late mortality, as supported by evidence from clinical trials. Despite the improved understanding of the complex pathological process of sepsis, much work remains to be completed in developing new drugs, combination therapies, and personalized treatments for sepsis.

Heparin, widely used in septic patients, has an unclear role in managing sepsis-associated DIC, as per a meta-analysis and an RCT [116,128]. Recombinant thrombomodulin, approved in Japan in 2008, outperformed heparin in a subgroup analysis of 80 sepsis-associated DIC patients and displayed a trend toward better 28-day survival rates in the SCARLET trial [116,125,129]. Hence, the Japanese guidelines recommend recombinant thrombomodulin for sepsis-associated DIC [130]. Extensive research on antithrombin has revealed its impact on severe sepsis, with a significant reduction in the 28-day mortality noted in patients with DIC [59,72]. Japanese guidelines, backed by a study from Tagami et al., recommend antithrombin alongside recombinant thrombomodulin for sepsis-associated DIC [130,131]. In comparison, the study illustrates the indeterminate efficacy of heparin for sepsis-associated DIC. Given the existing evidence, recombinant thrombomodulin and antithrombin appear promising but require further validation through multicentric RCTs. The potential benefits of combination therapy, specifically antithrombin with recombinant thrombomodulin, also need to be further studied.

## 11. Conclusions

In conclusion, sepsis-related DIC remains a challenging and life-threatening condition and has limited effective treatment strategies. Recent advances in understanding the pathological mechanisms of sepsis DIC and the development of new treatment methods offer some promise in improving patient outcomes. The role of anticoagulation therapy, including the use of heparin, recombinant activated protein C (APC), and antithrombin (AT), has been extensively studied; however, their efficacy and controversies still need to be addressed through further high-quality randomized controlled trials. Blood purification methods such as polymyxin B hemoperfusion and immunotherapy approaches, have the potential for treating sepsis DIC; however, further investigation is needed to assess their efficacy and safety. Multidisciplinary collaborations between clinicians, researchers, and healthcare organizations are essential for improving patient outcomes. Further studies are needed to find new therapeutic targets and understand the underlying mechanisms.

## Figures and Tables

**Table 1:** Review of definitions, pathogenesis, causes, clinical features, and diagnoses of sepsis and associated conditions.

Condition	Definition	Pathogenesis	Causes	Clinical Features	Diagnosis	Treatment	Reference
Sepsis	Life-threatening response to infection, causing organ dysfunction.	Dysregulated immune response, leading to inflammation.	Bacterial, viral, fungal infections.	Fever, chills, organ dysfunction.	Clinical symptoms, blood cultures.	Antibiotics, supportive care.	[4]
DIC	Widespread clotting in blood vessels.	Triggered by conditions like infections, trauma.	Sepsis, trauma, malignancies.	Bleeding, thrombosis, organ dysfunction.	Coagulation tests, low platelets.	Address cause, blood transfusions.	[2,3]
SIC	DIC subset linked to sepsis.	Interaction between inflammation and coagulation.	Prolonged PT, aPTT, decreased platelets.	Organ dysfunction, clot formation.	Focus on organ dysfunction.	Anticoagulants.	[2,3]
Septic Shock	Severe sepsis subset with high mortality risk.	Acute circulatory failure.	Elevated lactate, decreased platelets.	Hypotension, altered mental state.	Sepsis-3 definition.	Corticosteroids, immunomodulatory drugs.	[4]
SARS-CoV-2	Respiratory infection by SARS-CoV-2.	Virus targets ACE2 receptors; can cause ARDS.	SARS-CoV-2 transmission via droplets.	Respiratory symptoms, ARDS.	PCR, chest imaging, serological tests.	Symptomatic relief, antivirals.	[5,6,7,8]
Flaviviruses	Diseases from viruses like Zika, dengue.	Infection of immune cells, causing imbalanced response.	Mosquito-borne or direct contact.	Fever, rash, potential organ failure.	PCR, serological assays, culturing.	Supportive care, antivirals/antibiotics.	[7,9,10]

DIC: disseminated intravascular coagulation; SIC: sepsis-induced coagulopathy; SARS-CoV-2: severe acute respiratory syndrome coronavirus 2; PCR: polymerase chain reaction; ARDS: acute respiratory distress syndrome; ACE2: angiotensin-converting enzyme 2; PT: prothrombin time; aPTT: activated partial thromboplastin time.

**Table 2:** Understanding the molecular mechanisms, clinical manifestations, and therapeutic approaches of sepsis and related conditions.

Pathology	Molecular Mechanism and Pathways	Clinical Manifestations	Diagnostic Markers	Therapeutic Approaches	Relation to Sepsis-3 Controversy	Recent Research Findings	Reference
Sepsis	Systemic inflammatory response to infection, characterized by PAMPs release and activation of PRRs such as TLRs, triggering NF-κB and MAPK pathways. This results in cytokine storm and potential secondary infections.	Fever, tachycardia, dyspnea, hypotension, altered cognition, organ dysfunction.	Elevated proinflammatory cytokines, leukocytosis, thrombocytopenia, hyperlactatemia, organ dysfunction (SOFA score).	Broad-spectrum antibiotics, fluid resuscitation, vasopressors, corticosteroids, supportive care.	Sepsis-3 emphasizes organ dysfunction, potentially overlooking early sepsis without organ dysfunction.	Focus on early biomarkers and immune response role in sepsis progression.	[4,25,26]
DIC	Coagulopathy triggered by conditions, including sepsis. Characterized by widespread coagulation activation, microthrombi formation, organ dysfunction, ischemia, and bleeding manifestations.	Bleeding, purpura, petechiae, organ dysfunction.	Prolonged PT and aPTT, thrombocytopenia, increased FDPs, decreased fibrinogen.	Treatment of underlying cause, blood product transfusion, anticoagulants.	Sepsis-3 may not capture DIC complexity in sepsis due to organ dysfunction focus.	Exploration of DIC mechanisms in sepsis and potential coagulation cascade modulation.	[3,22,27,28,29]
SIC	DIC subset associated with sepsis. Characterized by coagulation activation, fibrinolysis inhibition, clot formation, and potential organ dysfunction.	Similar to DIC, including bleeding, purpura, petechiae, organ dysfunction.	Prolonged PT and aPTT, thrombocytopenia, increased D-dimer, decreased antithrombin III.	Treatment of underlying sepsis, potential anticoagulants.	Sepsis-3 may capture SIC patients but may not reflect underlying coagulation abnormalities.	Exploration of SIC mechanisms and potential therapeutic strategies, including anticoagulants.	[30,31]
Septic Shock	Sepsis subset with profound circulatory, cellular, and metabolic abnormalities. Characterized by persistent hypotension unresponsive to fluid resuscitation, requiring vasopressors.	Persistent hypotension, altered cognition, oliguria, tachycardia, dyspnea, cool and clammy skin.	Hyperlactatemia, thrombocytopenia, increased D-dimer, increased procalcitonin, organ dysfunction (SOFA score).	Vasopressors, antibiotics, fluid resuscitation, corticosteroids, supportive care.	Sepsis-3 includes septic shock as a subset with increased mortality. Criticized for complexity and need for laboratory results.	Exploration of septic shock pathophysiology and potential therapeutic strategies, including corticosteroids. and immunomodulatory drugs.	[4,25,26,32]
SARS-CoV-2	Virus enters host cells via ACE2 receptors, leading to viral replication and immune response activation. In severe cases, cytokine storm leads to severe inflammation and lung tissue damage.	Fever, cough, dyspnea, anosmia, fatigue, organ dysfunction in severe cases.	Positive RT-PCR for SARS-CoV-2, elevated proinflammatory cytokines, abnormal chest imaging.	Antivirals, corticosteroids, monoclonal antibodies, supportive care.	COVID-19 can lead to sepsis-like syndrome. Sepsis-3 may not capture unique aspects of COVID-19-related sepsis.	Focus on understanding severe COVID-19 pathophysiology, immune response role, and potential therapeutic targets.	[5,6,7,8,33,34,35]
Flaviviruses and Other Microorganisms	Different molecular mechanisms for host cell infection. Flaviviruses infect immune cells, leading to imbalanced immune response. Other microorganisms may produce toxins or virulent factors.	Symptoms vary, may include fever, rash, arthralgia, nausea, vomiting, diarrhea, cough, dyspnea.	Varies, may include positive culture or PCR, elevated proinflammatory cytokines, abnormal imaging.	Varies, may include antibiotics, antivirals, antifungals, supportive care.	Infections can lead to sepsis, but Sepsis-3 may not capture unique aspects of sepsis caused by these pathogens.	Exploration of pathogenesis of sepsis caused by these pathogens and potential therapeutic strategies.	[7,9,10,36,37,38]

**Table 3:** Microbiological features of bacteria known to cause sepsis and potentially progress to DIC, SIC, and septic shock, highlighting their impact on host defense.

Bacteria	GP	CA	SD	HE	SL	CP	SL	BF	RE
*Staphylococcus aureus*	+	+	+	+	+	+	+	+	FAN
*Coagulase-negative staph*	+	+	+	+	+	+	+	+	FAN
*Streptococcus pneumonia*	+	+	+	+	+	+	+	+	FAN
*Haemophilus influenza b*	+	+	+	+	+	+	+	+	MA
*Neisseria meningitidis*	+	+	+	+	+	+	+	+	FAN
*Klebsiella pneumonia*	+	+	+	+	+	+	+	+	FAN
*Enterococcus faecalis*	+	+	+	+	+	+	+	+	FAN
*Acinetobacter baumanii*	+	+	+	+	+	+	+	+	A
*Escherichia coli*	+	+	+	+	+	+	+	+	FAN
*Salmonella enterica*	+	+	+	+	+	+	+	+	FAN
*Shigella dysenteriae*	+	+	+	+	+	+	+	+	FAN
*Citrobacter freundii*	+	+	+	+	+	+	+	+	FAN
*Serratia marcescens*	+	+	+	+	+	+	+	+	FAN
*Proteus mirabilis*	+	+	+	+	+	+	+	+	FAN
*Pseudomonas aeruginosa*	+	+	+	+	+	+	+	+	FAN
*Bacteroides fragilis*	+	+	+	+	+	+	+	+	OAN

Abbreviations: Features > GP (Glutathione Peroxidase); CA (Catalase); SD (Superoxide Dismutase); HE (Hemolysins); SL (S-layer); CP (Capsule); SL (Slime Layer); BF (Biofilm); RE (Respiration: FAN (Facultative Anaerobic Bacteria); MA (Micro Aerobic Bacteria); A (Aerobic Bacteria); OAN (Obligate Anaerobic Bacteria); "+" positive result for the tests/characteristics. Note: These bacteria can cause sepsis, and not all cases of sepsis will progress to DIC, SIC, or septic shock. The progression of the disease can depend on a variety of factors, including the individual’s immune response and underlying health conditions.
